# Beyond Economics! The (Evolving) Role of Law in the Eradication of Extreme Poverty

**DOI:** 10.1007/s41134-023-00247-2

**Published:** 2023-05-20

**Authors:** Augustine Edobor Arimoro

**Affiliations:** 1 Roehampton Law School, Faculty of Business and Law, University of Roehampton London, London, UK; 2 Centre for Comparative Law in Africa, University of Cape Town, Cape Town, South Africa

**Keywords:** Extreme poverty, SDGs, Human rights, Sub-Saharan Africa, Law and justice

## Abstract

Extreme poverty is a complex and multifaceted challenge that cannot be solely addressed through economic interventions. Traditional economic indicators, such as GDP, do not fully capture the realities of vulnerable populations who often experience discrimination and social exclusion. This has legal and human rights implications, particularly in regions such as Sub-Saharan Africa where extreme poverty is concentrated. In light of these concerns, this article critically examines the existing literature on poverty economics and law and presents an analysis of key data. Ultimately, the article argues for a comprehensive approach that prioritises law and justice as crucial components of efforts to achieve target 1 of the United Nations’ Agenda 2030 for Sustainable Development. This approach should entail the establishment of legal frameworks that promote accountability for political actors and protect the rights of the poor.

## Introduction

Economists often link economic growth with poverty. Economists argue that as the economy grows, there would be a corresponding reduction in the rate of poverty in any jurisdiction (Zhu et al., 2022). Furthermore, economists tend to measure “poverty, prosperity and growth” in monetary terms and in consonance with the per capita income of the citizens of a given country (Roser, 2021). Ironically, however, linking prosperity with per capita gross domestic product (GDP) does not necessarily mean that it reflects most of the population’s wealth or poverty level. For example, based on recent data available from the World Bank in 2021 (The World Bank, 2021), India is the sixth largest economy in the world with a GDP of US $2.66 trillion, while Australia with a GDP of US $1.32 trillion. From this perspective, India is more prosperous than Australia. The reality is that 25% of the population of India is poor (Aayog, 2022). On the other hand, less than 12% of the population of Australia is in poverty as of 2020 (Simon-Davies, 2022). While this paper does not seek to propose a new indicator for determining the wealth or poverty of a state, this opening analysis suggests that when discussing the poverty of a state, GDP which is often a factor for economists, is perhaps not the best demarcation between prosperity and poverty. It is a truism that poverty reduction may not keep pace with the perceived accelerated economic growth in countries with a concentration of extreme poverty levels.

**Fig. 1:**
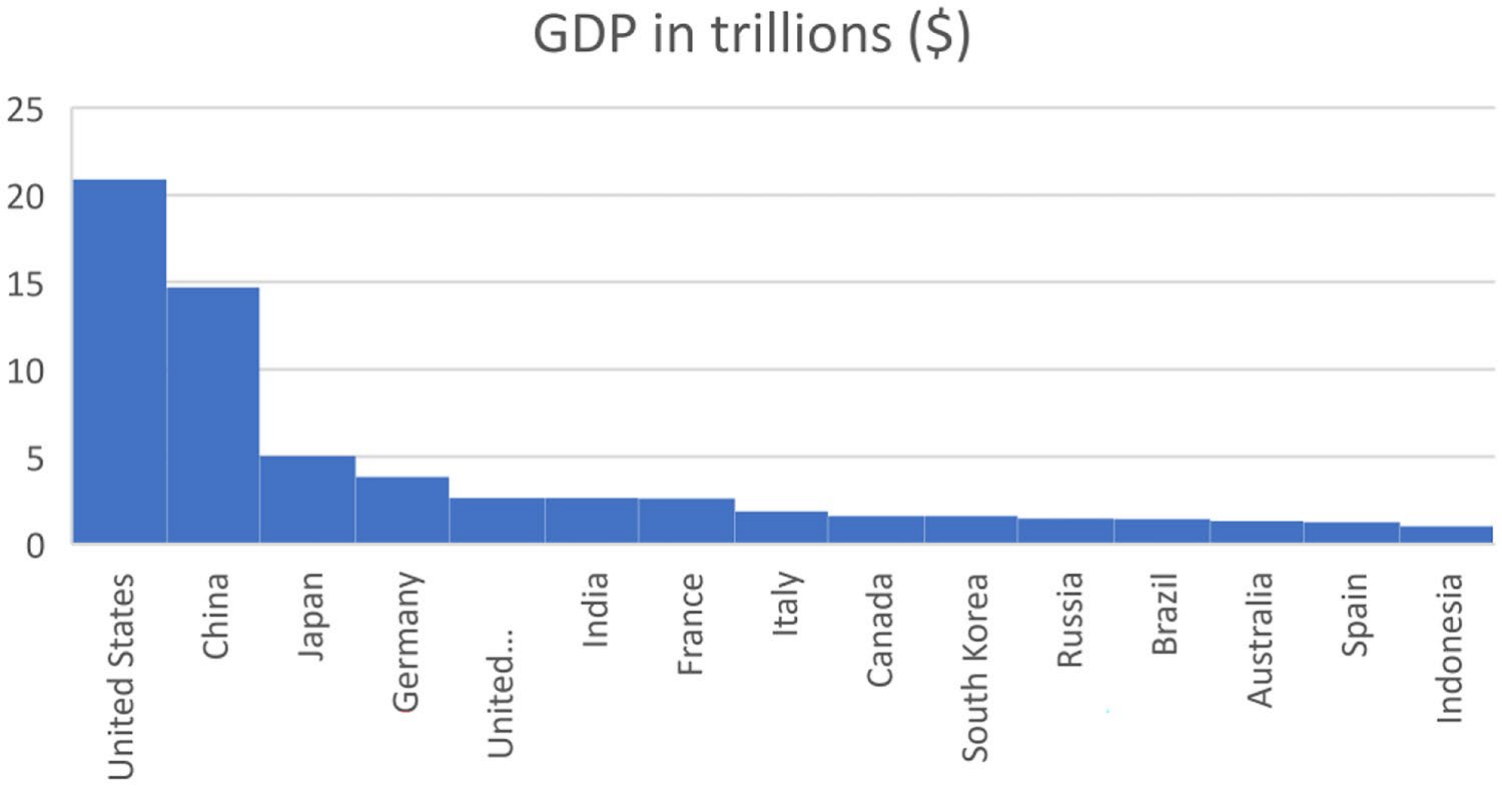
Top 15 countries by GDP in 2022 — author, data from the World Bank

Figure 1 below shows the top 15 countries of the world by GDP figures in trillions in 2022. While a high GDP may reflect that a country is prosperous, it may mispresent the level of poverty experienced by the ordinary citizens of that country.

This article aims to discuss the (evolving role) of the law and legal systems around the world in the quest to eradicate extreme poverty. Although economists have and do play a core role in poverty reduction and eradication (Si et al., 2020), there is a key role for law and legal systems that should be exploited in countries with high poverty rates. There is a need, therefore, to think of poverty in the legal sense (Hernandez, 2020). The overarching question that this paper seeks to deal with is this: how might the law and legal systems promote prosperity in regions with high levels of poverty around the globe? Following the above, the article considers poverty as a phenomenon in the legal sense.

More so, the article argues that eradicating poverty is ambitious. It demonstrates why the UN’s approach to eradicating “extreme poverty and substantially reducing moderate poverty by 2030” (FAO, 2022) is achievable. A review of the efforts since the implementation of the SDG programme is essential given that the world is in the final decade of the United Nations (UN) 2030 Agenda for SDGs. Ending poverty in all its ramifications is the first of the 17 SDGs of the 2030 Agenda for Sustainable Development. Interestingly, the SDGs’ main reference to combatting poverty is set out in target 1 A:

Ensure significant mobilization of resources from a variety of sources, including through enhanced development cooperation, to provide adequate and predictable means for developing countries, in particular, least developed countries, to implement programmes and policies to end poverty in all its dimensions.

With the above postulation in view, this article evaluates three different law-centred approaches to dealing with the extreme poverty problem in the world. The study shows that the biggest concentration of people living in extreme poverty lives in Sub-Saharan Africa. As such, the article makes a case for an SSA-centred approach along the lines of the legal socio-economic theory to eliminate extreme poverty and social inequalities.

## Conceptual Analysis

The United Nations agreement on SDGs underscores an ambition to eliminate extreme poverty by including an equality goal and by making sustainability the core of the global body’s agenda for 2030 (Stewart, 2015). It is therefore not a surprise that the first out of 17 goals following the recommendations of the Open Working Group is no poverty by the year 2030. Tracking the progress made so far in the quest to eliminate poverty requires an assessment of extreme poverty using specific indices.

Like other concepts known to social scientists, there is no universal definition of poverty (Dagunga et al., 2021). As pointed out above, the monetary or GDP approach to analysing poverty is deficient as it does not capture the intensity of poverty. For example, Dagunga et al. note that in 1977, a poor fellow in rural Kenya was asked to describe poverty and the man’s response were as follows:

Do not ask me what poverty is because you have met it outside my house. Look at the house and count the number of holes. Look at the utensils and clothes I am wearing. Look at everything and write what you see is poverty (Dagunga et al., 2021).

The above supports the view that there should be other indicators to measure poverty beyond GDP and monetary measures. To O’Boyle, the question, “What is poverty?” should be preceded by “What does it mean to be poor?” (O'Boyle, 1999). The answer to this second question potentially offers a better measure for determining poverty across the nations of the world. This is in contradistinction to merely rely on a poverty measurement index. For example, the new World Bank standard for measuring extreme poverty is a monetary measure and the international poverty line is set at US $2.15 per person per day (The World Bank, 2022). According to the World Bank, anyone living on less than US $2.15 a day is living in extreme poverty (The World Bank, 2022). Those living in extreme poverty are estimated to be around 648 million as of 2019 (The World Bank, 2022). Interestingly, the COVID-19 pandemic and the current war in Ukraine have impacted whatever progress the world has made in the elimination of extreme poverty. With a cost-of-living crisis envisaged in the coming months around the world, there is the likelihood that it will aggravate poverty conditions across the world (Swinford, 2022). The number of people living in extreme poverty increased by an estimated 50 million because of the pandemic and global economic meltdown (Suckling et al., 2021).

**Fig. 2:**
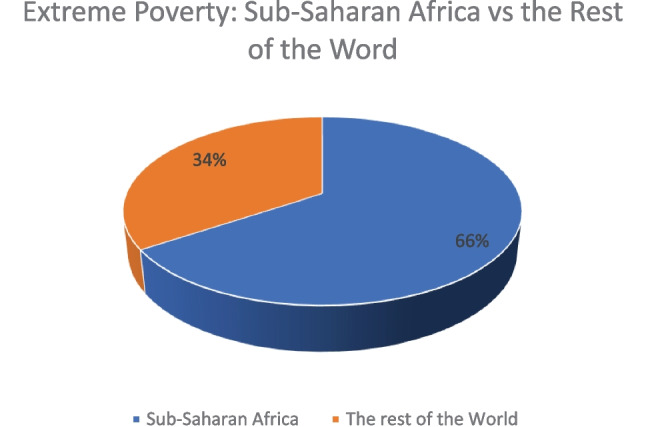
Extreme poverty: Sub-Saharan Africa vs the rest of the world: source — author with figures from development initiatives

Figure 2 represents the distribution of people living in extreme poverty across the world. Sub-Saharan Africa (SSA) has the largest share of people living in extreme poverty estimated to be around 458 million compared to the rest of the world with 240 million people. The data reviewed for this study shows that 26 countries in SSA have seen the number of people living in extreme poverty increase significantly between the years 2010 and 2020. The largest increases are visible in Angola (9.4 million), Congo DRC (8.8 million), and South Sudan (7 million) (Suckling et al., 2021). It follows that dealing with extreme poverty must require a strategy that addresses the SSA extreme poverty problem being the region with the greater share of people living in extreme poverty. A starting point would be to interrogate how some SSA countries have been able to ensure a reduction in the number of people living in extreme poverty within the same period. For example, Ethiopia (12 million), Guinea (3.4 million), and Nigeria (4.5 million) (Suckling et al., 2021). However, it remains to be noted that the determinant of extreme poverty increase/reduction above is based on economic measurements which may not necessarily reflect the true poverty situation of the people.

**Fig. 3:**
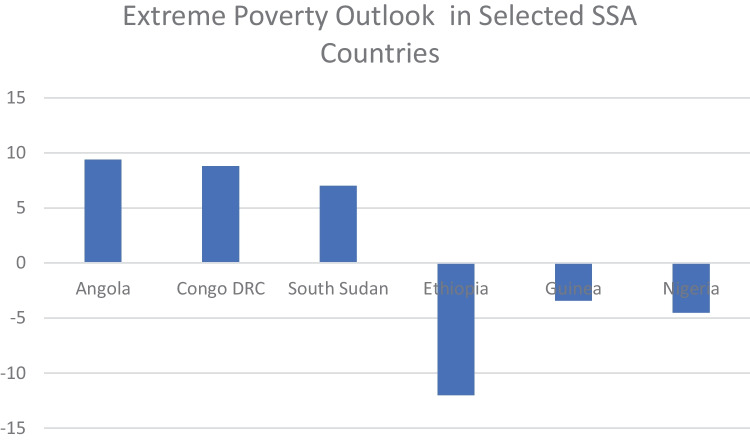
Extreme poverty outlook in selected SSA countries: source — author with data from the World Bank

Figure 3 below shows the number of people who have either moved into extreme poverty or moved out of extreme poverty in selected African countries. Regrettably, Angola and Congo with the largest share of people who have moved into extreme poverty between 2010 and 2020 are countries endowed with significant human and natural resources. Angola is the largest producer of crude oil in Africa (Hollands, 2022); Congo DRC has an abundance of natural wealth including crude oil, hydro energy, diamonds, and gold (United Nations, 2003). Of note, rather than these natural resources being applied to eradicate extreme poverty, they mostly count towards indicators that show national wealth even though citizens are impoverished and exposed to human rights abuses (United Nations, 2003).

Another deficiency in measuring poverty in terms of monetary value is the fact that poverty transcends an economic reportage. A better understanding and reporting of extreme poverty should be expanded to include other measures such as inequality and vulnerability (Suckling et al., 2021). To achieve the goal of “no poverty by 2030,” there is a need for a holistic approach to eliminating extreme poverty and reducing poverty. To this end, the contribution of lawyers and the justice system cannot be overemphasised.

Reflecting on what constitutes poverty remains a key area of consideration for this article. According to the UN:

Poverty entails more than the lack of income and productive resources to ensure sustainable livelihoods. Its manifestations include hunger and malnutrition, limited access to education and other basic services, social discrimination and exclusion, as well a lack of participation in decision-making (United Nations, 2020).

The above position is a better way to describe poverty rather than limiting it to the amount of money a person spends a day. However, this definition does not help with determining what amounts to extreme poverty. Thankfully, the UN 2030 Agenda recognises that poverty reduction approaches shall not only focus on reducing monetary poverty but also they must go in *pari passu* with strategies to build economic growth while at the same time addressing a range of needs such as education, health, social protection, and job opportunities. In addition, measures taken will address climate change and environmental protection.

## Extreme Poverty in Sub-Saharan Africa

To deal with the problem of extreme poverty in SSA, it is pertinent to understand poverty in the context of the region. There are 49 countries in SSA. Significantly, 34 of these 49 countries are considered least developed countries (LDCs) (The Ministry of Foreign Affairs (Japan), 2014). In this section of the paper, World Bank poverty data on SSA is analysed. The data studied is from the recent report on poverty published by the World Bank in 2020. The data is based on surveys conducted (The World Bank Group, 2020).

The data in focus in this section are the percentage of the population in the respective SSA countries that live below the international poverty line (monetary poverty) and where they can be found. This has been categorised into urban/rural areas. Table 1 below presents a snapshot of the poverty spread in SSA.

**Table 1:** A glance at poverty in Sub-Saharan Africa: source — author with data from the World Bank

Country	Monetary poverty (%)	National poverty line (%)	Urban population (%) of persons living in poverty	Rural population (%) of persons living in poverty
Angola	47.6	36.6	32	73
Benin	49.5	40.1	36	60
Botswana	15.7	19.3	31	57
Burkina Faso	43.7	40.1	N/A	47
Cabo Verde	3.2	35	29	59
Cameroun	23.8	37.5	10	61
CAR	66.3	62	51	75
Chad	38.4	46.7	14	44
Comoros	17.6	42.4	N/A	N/A
DRC Congo	76.6	63.9	56	89
Congo	37	40.9	20	71
Cote d’Ivoire	28.2	46.3	16	40
Eswatini	28.4	58.9	9	49
Ethiopia	30.8	23.5	13	35
Gabon	3.4	33.4	N/A	N/A
The Gambia	10.1	48.6	2	20
Ghana	13.3	23.4	3	24
Guinea	35.3	55.2	13	46
Guinea-Bissau	67.1	69.3	55	75
Kenya	36.8	36.1	17	48
Lesotho	26.9	49.7	N/A	N/A
Liberia	40.9	50.9	21	64
Madagascar	77.6	70.7	40	85
Malawi	70.3	51.5	25	81
Mali	49.7	41.1	13	60
Mauritania	6	31.0	2	10
Mauritius	0.2	10.3	N/A	N/A
Mozambique	62.9	46.1	41	73
Namibia	13.4	17.4	6	10
Niger	44.5	44.5	N/A	85
Nigeria	53.5	46.0	38	62
Rwanda	55.5	38.2	26	62
Sao Tome & P	34.5	66.2	35	33
Senegal	38	46.7	12	58
Seychelles	1.1	39.3	N/A	N/A
Sierra Leone	40.1	52.9	19	57
South Africa	18.9	55.5	N/A	63
South Sudan	42.7	82.3	15	48
Sudan	16.2	46.5	5	22
Tanzania	27.6	14.9	29	58
Togo	49.8	55.1	23	69
Uganda	41.7	21.4	45	78
Zambia	57.5	54.4	25	81
Zimbabwe	33.9	70	N/A	N/A

A glance at Table 1 above shows clearly that most of the population living below the monetary poverty line (or in extreme poverty) in countries in SSA can be found in rural areas. The exception is Sao Tome and Principe. It, therefore, means that to deal with the challenge of extreme poverty in SSA, policies that focus on rural development should be key in addressing the challenge of extreme poverty.

It is worthy of note that although there is a large concentration of people living in extreme poverty in SSA, a few countries in the region are not as burdened by the high population of persons living in extreme poverty as do the majority. For example, Mauritius, Mauritania, Gabon, and Cabo Verde have a low percentage of their population living in extreme poverty. The data in Table 1 underscores the fact that the size of the economy of a country may not be the key factor in determining whether the population of that country lives in prosperity or not. For example, although Nigeria with a GDP of about 441.5 billion dollars in 2021 and South Africa with a GDP of about 418 billion dollars in 2021 are the first and second largest economies in Africa respectively (Kamer, 2022), it does not necessarily reflect in the number of the population living in extreme poverty.

Furthermore, it is pertinent to explore the causes of extreme poverty levels in SSA. Most of the countries in the region are rich in human and natural resources but this does not reflect in society due to inequalities (Ali & Thorbecke, 2000). Again, while the economies of these countries grow, a constant paradox is the fact that a large part of the population of SSA remains trapped in economic poverty (Kabuya, 2015). SSA has been identified as the reason behind the global extreme poverty reduction (Schoch & Lakner, 2020). The main causes of poverty in SSA include and are not limited to poor basic infrastructure, lack of access to jobs and adequate sources of livelihoods, conflict and instability, inequality and social injustice, and lack of personal safety nets (Abulencia, 2022).

## A Human Rights Approach

Can a human rights approach provide a better measurement as well as serve as a mechanism for eradicating poverty in SSA? The human rights-based approach (HRBA) proposes that human rights standards form the core of planning, policy, and practice in the context of the elimination of extreme poverty and reduction of poverty (European Network of National Human Rights Institutions, 2019). There is a strong argument that poverty should be measured as a multidimensional phenomenon. This approach favours combining economic factors and lack of access to the goods or services essential to “the enjoyment of human rights such as housing, education, healthcare, food, decent work and social security” (European Network of National Human Rights Institutions, 2019). Furthermore, proponents of the HRBA approach hold that poverty and poverty elimination strategies should not be construed in a narrow sense, i.e., in monetary terms.

A weakness of the HRBA is that human rights scholars tend to focus on vulnerability as a key indicator rather than the broad multidimensional approach (Sano, 2020). Be that as it may, any analysis on poverty elimination or reduction that does not have at its core, a real transformation in the standard of living of ordinary people, is a transformation only reflected on paper. Indeed, poverty is the “gravest human rights challenge facing the world today”. Poverty, therefore, is not only an economic problem. It is also a human rights problem that requires human rights solutions. In the same vein, there is a link between poverty, equity, and health which must not be waved aside. Therefore, it is incumbent on States around the world to implement strategies that will use human rights norms to assert the standards for optimal health and prohibit discrimination based on gender, disability, race, national origin, or religion (Braveman & Gruskin, 2003).

### International Human Rights Norms

The position of the UN, among others, is that the adoption of an HRBA in tackling extreme poverty should be anchored on the norms and values enunciated in international human rights law (United Nations, 2012a, b). The Human Rights Council of the UN on 27th September 2012 adopted by consensus the Guiding Principles on extreme poverty and human rights. The Guideline encapsulates the goals of the Council in the following words:

A human rights approach respects the dignity and autonomy of persons living in poverty and autonomy of persons living in poverty and empowers them to meaningfully and effectively participate in public life, including the design of public policy, and to hold duty bearers accountable. The norms set out in international human rights law require that States take their international human rights obligations into account when formulating and implementing policies affecting the lives of persons living in poverty.

Although persons living in extreme poverty cannot simply be reduced to a list of vulnerable groups, discrimination and exclusion are among the major causes and consequences of poverty. Persons living in poverty often experience disadvantage and discrimination based on race, gender, age, ethnicity, religion, language or another status. Women frequently encounter greater challenges in accessing income, assets and services and are particularly vulnerable to extreme poverty, as are children, older persons, persons with disabilities, migrants, refugees, asylum seekers, internally displaced persons, minorities, persons living with HIV/AIDS and indigenous peoples (United Nations, 2012a, b).

It is not out of place, to reiterate, at the risk of repetition that a human rights approach presents a picture of vulnerable people as people needing protection from the state rather than an economic picture of the money in a person’s pocket.

Indeed, the Council identifies that discrimination is both a cause and a consequence of poverty (United Nations, 2012a, b). Any plans to eliminate extreme poverty without dealing with problems of discrimination against persons of any kind would be futile. As such, it is incumbent on States to ensure that those living in poverty are equal before the law and are entitled to equal protection and benefit of the law. States must make efforts to repeal laws that discriminate against certain people groups. For example, in SSA, several countries have existing colonial era-inspired anti-gay laws that sponsor homophobia (Arimoro, 2021). Countries like Nigeria, Ghana, Kenya, and Uganda have strict laws with severe punishments for persons convicted of offences against the order of nature, a colonial coinage (Arimoro, 2021). A former Nigerian president, Dr Goodluck Jonathan in 2014 signed into law, a Bill which makes it a criminal offence to enter same-sex relationships, to lobby, advocate for, or support LGBT rights (International Service for Human Rights, 2014). The Uganda Parliament in 2021 passed a controversial sexual offences Bill which further criminalises same-sex relationships and sex work (Okiror, 2021). Similarly, some African States including Nigeria have signed treaties for the protection of persons living with disabilities and have been slow in domesticating or giving effect to laws that protect such persons (Arimoro, 2019b). The discrimination against women in some parts of SSA aggravates poverty levels and is an area that requires state action, not just in finely crafted policies but demonstrated by the political will of the State (Ekhator, 2019).

It is argued, and rightly so, that lack of awareness of human rights by the public correlates with poverty (Mubangizi, 2005). It should be noted that some of the rights which are essential to the elimination of extreme poverty are mostly socio-economic rights and as a result, non-justiciable in several developing regions of the world including the SSA. The fact that socioeconomic rights have been constitutionalised in some States should be a reason why the citizens should demand that political leaders implement policies that would ensure that these rights are enjoyed by citizens. For example, South Africa to some extent presents a working model that can be mirrored by other SSA countries in the realisation of socio-economic rights (Shehu, 2013).

An HRBA to tackle extreme poverty must be based on international human rights norms which aim to tackle poverty (Mubangizi, 2005). To this end, the United Nations Committee on Economic, Social and Cultural Rights (UNCESCR) states as follows:

Anti-poverty policies are more likely to be effective, sustainable, inclusive, equitable and meaningful to those living in poverty if they are based on international human rights.

Following the above, an approach to ending extreme poverty will mean relying on all international human rights instruments that constitute the International Bill of Rights as well as domestic law that bring home those rights to the different States of the world. Thus, for example, a starting point will be Article 17 of the Universal Declaration of Human Rights (UDHR). The Article provides in (1) that everyone has the right to own property alone as well as in association with others and further provides in (2) that no one shall be arbitrarily deprived of his property. Article 22 of the UDHR further provides for the right to social security. Also, Article 23 of the UDHR makes provision for the right to work and just and equal pay without discrimination. These rights can serve as the core basis for a strategy for eliminating extreme poverty. Furthermore, Article 25(1–2) of the UDHR gives credence to the right to proper living standards in the following words:

1. Everyone has the right to a standard of living adequate for the health and well-being of himself and his family including food, clothing, housing and medical care and necessary social services, and the right to security in the event of unemployment, sickness, disability, widowhood, old age or other lack of livelihood in circumstances beyond his control.
2. Motherhood and childhood are entitled to special care and assistance. All children, whether born in or out of wedlock, shall enjoy the same protection.

An adequate standard of living should well be above the level of poverty. It behoves the States of the world to ensure that policies are not only designed but also implemented to ensure that citizens have adequate healthcare, food, clothing, and housing.

The International Covenant on Economic, Social and Cultural Rights (ICESCR) is in sync with the UDHR and in fact, lays down in more detail rights that directly relate to eliminating extreme poverty. These rights have been incorporated in the guidelines for the integration of human rights into poverty reduction strategies, designed by the Office of the Commissioner for Human Rights (Mubangizi, 2005). The relevant ICESCR highlights the right of everyone to the enjoyment of just and favourable conditions of work which include fair wages, decent living, safe and healthy working conditions, and equal opportunities for promotion and leisure (including holidays).

Other international human rights instruments provide a clear framework for States to apply in their national strategies to eliminate extreme poverty. States are obligated to respect these norms (United Nations, 2022a, b). They include and are not limited to the Convention on the Elimination of All Forms of Discrimination against Women (CEDAW), the International Convention on the Elimination of All Forms of Racial Discrimination (ICEAFRD), the International Covenant on Civil and Political Rights (ICCPR), Convention on the Rights of the Child, International Convention on the Protection of the Rights of All Migrant Workers and Members of Their Families (CMW), and the Convention on the Rights of Persons with Disabilities (CRPD).

Another important instrument is the United Nations Declaration on the Rights of Peasants and Other People Working in Rural Areas (UNDRPPRA). This instrument will help improve living conditions in rural communities. If this instrument is adopted and implemented in the Global South, it will make issues regarding land ownership, working in rural areas and improvement of biodiversity workable. In the same vein, where there is expropriation of land belonging to rural dwellers, the public authorities should ensure that adequate compensation is paid (Arimoro, 2019b).

### Guidelines for the Integration of a Human Rights Approach in Poverty Reduction Strategies

It is important to mention that the Office of the United Nations High Commissioner for Human Rights designed the guidelines for a human rights approach to poverty reduction strategies in 2001 (United Nations, 2022a, b). It is submitted that countries experiencing a high level of extreme poverty should exercise the political will to implement these guidelines in the next few years before the target date of 2030 goals.

The UN’s Guide sets out seven principles for National Human Rights Institutions (NHRIs) to advance an HRBA to poverty reduction and measurement. The principles include accountability, equality, and non-discrimination, participation, data disintegration, using plural methodologies, measuring the non-take-up of rights and safeguarding data (United Nations, 2022a, b). These principles are clearly explained in the document. It is not the aim of this writer to reproduce the principles in this article.

In concluding this section, it is relevant to state that although poverty is most times depicted as a lack of necessities, there remains an approach to better contend with poverty if it can be viewed from the lens of international human rights. As Rukooko (2010) puts it, there is a nexus between the embarrassing poverty situation in Africa and human rights. Furthermore, although this article has identified that there is a huge concentration of people living in extreme poverty in SSA, it remains to be stated that extreme poverty is not unique to the Global South alone. For example, Farley states that the European Court of Human Rights (ECHR) acknowledges that extreme poverty correlates with human rights violations (Farley, 2022). Again, the recent floods in Houston neighbourhoods in the USA saw low-income neighbourhoods filled not only with water but also with heavy chemicals which is a legacy of environmental racism that permitted the existence of toxic industries in neighbourhoods of people of certain extractions (Hughes, 2020).

## A Development and Justice Approach

One of the key dimensions of poverty is a lack of access to justice (Biebesheimer & Chapman, 2012). Access to justice is a fundamental principle of the rule of law (United Nations, 2022a, b). Poverty is a two-edged sword. It “fosters and it is itself a consequence of injustice” (Goertzen & Neilsen, 2022). The legal system can play and should play a leading role in supporting the elimination of extreme poverty around the world. The justice system must ensure that poor people have access to the right mix of rights and remedies (United Nations, 2009). People who live in poverty encounter varying challenges as far as access to justice is concerned. They are often denied the opportunity to challenge crimes or human rights violations committed against them (Donald & Sepulveda, 2015).

The rule of law is vital if there must be sustainable economic development around all the countries of the world in line with the 2030 SDGs Agenda. It is not enough to have laws protecting the rights of vulnerable people and the laws remain sleeping or for want of the resources to ensure that the laws work. In simple terms, legal protection without the requisite resources to access the legal safeguards and/or remedies amounts to being denied the protection in the first place. Sadly, it is noted that around four billion people in the world do not enjoy legal protection (Open Society Foundations, 2019). Interestingly, the access to justice approach would afford a linkage between goal 1 and goal 16 of the SDGs. This is more so as goal 16 targets tackling violence, promoting accountability and transparency as well as a call for access to justice and the promotion of the rule of law at all levels (Open Society Foundations, 2019).

### Legal Awareness

Making information about access to justice available to people living in poverty is a key part of the access to justice approach (Carmona & Donald, 2015). All countries that have agreed to the SDGs have pledged to “promote the rule of law at the national and international levels, and ensure equal access to justice for all,” as part of SDG 16 (OECD, 2016). It is the responsibility of States to sensitise citizens about their rights and how to enforce those rights. One way to achieve this in the mid-to-long term is the inclusion of human rights and access to justice as a part of the curriculum in schools. Furthermore, countries without national orientation agencies should establish one that would focus on sensitising citizens to their rights. The works of NGOs in this regard especially in the SSA context deserve commendation. For example, the efforts of pro-rights groups supporting the decriminalisation of same-sex relationships as well as pro-disability rights have played in ensuring the protection of vulnerable persons. The signing into law of the Disability Act in 2019 by the Government of Nigeria demonstrates the effect of pressure that rights groups can bring on political leaders (Arimoro, 2019b).

States in the Global South could adopt a strategy that will see to the establishment of legal information centres like the UK-styled Citizen’s Advice Bureau. It is key that citizens of any given country get the relevant information they need free of charge. In some countries in the SSA region, widows in rural areas have been denied the opportunity of farming on their deceased spouse’s land, especially where they refuse to marry a relative of their deceased spouse (Human Rights Watch, 2017). In Zimbabwe, for instance, a widow was only able to take back her inheritance and resumed farming after the intervention of the Legal Resources Foundation (LRF), a legal aid organisation because her neighbour told her about the LRF (Human Rights Watch, 2017).

### Strategies to Promote Access to Justice

People in rural areas must have easy physical access to the courts and police stations. Often court houses and police posts are in urban areas in developing economies. This is a deprivation for those that live in rural areas. Judges, lawyers, and court administrative staff should be incentivised to work in rural areas.

While lawyers should be encouraged to take on some pro bono work annually, the states must not shy away from establishing and funding a legal aid system to cater to the needs of the less privileged in society. A significant proportion of persons living in extreme poverty are also persons living with disabilities. States must find to ensure the inclusion and accommodation of persons with disabilities as far as access to justice is concerned (Arimoro, 2019a). Sign language interpreters should be trained to work with the justice system.

To avoid discrimination against persons who cannot afford court filing fees, the justice system must provide a means for accommodating persons within certain income thresholds that cannot afford to pay court filing fees. Governments must take special measures to prohibit discrimination against the poor by way of eliminating high court filing fees (United Nations, 2009). It has been argued that citizens should be able to challenge by judicial review excessive court fees (The Honourable Justice McCloskey, 2018). The question that follows is this. How can a citizen who wants to challenge court fees to apply to the court without paying the fees? Thus, it may be more effective to put pressure on political representatives to do right by the people. There has been another argument supporting traditional mechanisms for the resolution of disputes as this is much cheaper than the formal court system. While this approach may ensure quick dispensation of justice, it should only be provided as a means of resolving disputes like the modern concept of arbitration. Where parties accept that their dispute should be settled by a chief or by a council of elders in purely civil matters, this should be encouraged. However, it must be clear that where a party is not satisfied by the ruling of the informal traditional court, then that party can approach the formal courts for a remedy. No informal traditional court setting should hear or determine a criminal case.

Civil society and rights groups should be encouraged by the international community in their work, especially in SSA. Civil rights groups should be accorded the right of legal status to appear in court on behalf of vulnerable persons. Any such law, for example, a strict regime that is built on the *locus standi* principle must be abolished in the interest of the vulnerable members of the society (The United States Institute of Peace, 2022).

### A Social Welfare Approach to Eliminating Extreme Poverty

Following the above, SSA countries can adopt a social welfare approach in dealing with extreme poverty. For example, a welfare system should be introduced to assist people living in extreme poverty (Joseph, 2021). Relying on the extended family system as the main source of welfare in SSA countries as the world approaches, the 2030 target year is not sustainable. There has been slow commitment and lack of political will in ensuring that the needs of those living in extreme poverty are catered to in SSA for many years (Carbone & Pellegata, 2017). Again, it is argued that privatisation has negatively impacted social welfare in SSA (Okeahalam & Mukwena, 2000). The privatisation of many public-owned corporations and services has put the price of some utilities and services out of the reach of the poor. Given the level of extreme poverty in the region, governments must put in place realistic welfare systems besides the occasional payment of handouts to poor families.

The United Nations has proposed social protection for vulnerable persons in society in the quest to eliminate extreme poverty (United Nations, 2018). This would involve removing barriers that persons with disabilities face (Arimoro, 2019a, b, c), ensuring adequate disability benefits as well as ensuring that older persons receive their pensions (Ralston et al., 2019).

### A Socio-Economic Conditions Approach Founded on the Law

It has been established already in this article that most of the people living in extreme poverty in the world live in SSA. As much as eliminating extreme poverty is a global goal, it is necessary for governments in the SSA region to take proactive steps to eliminate extreme poverty in the region. To do this, SSA must look inwards for solutions rather than rely on neo-colonial/liberalist approaches that seldom work in the SSA region. It is submitted that while the SSA applies the first two approaches, viz, human rights and the justice approach, the template used must suit the socio-economic environment.

Professor YS Lee in his seminal work entitled “General Theory of Law and Development” (Lee, 2017) expanded the socioeconomic conditions theory of law and development. Lee proposes templates which are in sync with the regions that they are meant to develop rather than for example, trying out neo-liberal Western ideas in the developing world which had been the hallmark of the erstwhile law and development school. This theory aligns with Professor Ordor’s earlier postulation that law and development should adopt a multidimensional approach (Ordor, 2015). The theory has been tested in later works (Arimoro, 2022; Ghebremusse, 2019).

It is not out of place to begin by asking the vexed question: what is the theory of the socio-economic conditions? Law and development scholars make a case that the law is an instrument for development and that law can be used as a tool to give effect to policies that spur economic growth and raise the standard of living of citizens in any given state (Mcauslan, 2007). Traditional law and development theories have been criticised for not being pro-Global South. Hence, the vacuum for law and development scholarship that is “Global South-centric.” For example, Garcia argues that there is a new reality which is the need for developing economies to reduce the IMF influence and explore development that echoes the needs of developing economies (Garcia, 2016).

Unfortunately, traditional “law and development” was tied to the financial and development assistance provided by national development agencies and international financial institutions (IFIs) (Garcia, 2016). This approach has not helped the impoverished people of the Global South and especially the SSA region. It is in this wise that scholars like Lee (2017) and Ordor (2015) champion a new approach that considers the needs of the Global South when international policies and domestic strategies are put in place. One fallacy of neo-liberal ideas is the failure to recognise that developing economies need a different kind of strategy from the ones adopted in the Global North. The argument that wealthy countries have responded to the institutional problems in the Global South through the efforts of the bilateral aid agencies and multilateral development banks on reforming the public sector in developing agencies is weak (Parks & Buntaine, 2017). International development organisations have played a role in the problem of real underdevelopment in the Global South. For example, Parks and Buntaine (2017) note as follows:

…international development organizations contribute to this problem by encouraging many of their developing country counterparts to select performance targets that measure institutional forms rather than institutional functions. Developing countries that are eligible to receive grants and below-market-rate loans have strong incentives to meet targets because they must do so to access financing. As a result, they often select easy targets that allow them to declare victory instead of more difficult targets that measure whether institutions are solving problems. Countries that are not eligible to receive this type of concessional financing, by contrast, have no such incentive and are more likely to choose difficult targets that are more relevant to the lives of their citizens.

The argument here is that unless realistic standards align with the needs of the citizens of the Global South and especially SSA (where there is the largest concentration of people living in extreme poverty are found), more and more pyrrhic victories would be declared by the governments using questionable standards of measurements which suits the fancies of international organisations.

#### Supporting Frameworks that Match the Needs of the Global South

Lee argues, and rightly so, that law and development should take into cognisance local variances (Lee, 2017). It follows that there cannot be a one-size-fits-all approach for the entire globe (Lee, 2018). It is okay if the goal of the UN is to ensure that extreme poverty is wiped out by the year 2030. However, there should be room to apply multidimensional approaches to fix the problem (Ordor, 2015).

According to Lee, “Law may not be effective if it does not conform to the socioeconomic conditions on the ground” (Lee, 2017). He further describes socioeconomic conditions to be the range of social, political, economic, and cultural conditions that are the prerequisite for the successful operation of law (Lee, 2017). Socioeconomic conditions would include the social and religious norms prevalent in any given society. Lee cites as an example a statute that authorises the charging of interest by banking and non-banking financial institutions to enable financial growth may not be effective in an environment that considers charging interest for any service unethical (Lee, 2017). Charging interest or *riba* is considered unlawful (*haram*) in Islam (O'Sullivan, 2020).

Lee (2017) illustrates how several developing countries including Vietnam, Cambodia, Myanmar, and Bangladesh have shown interest in adopting certain Korean laws. This supports the notion that countries with similar traditions or cultures may well adopt templates from their peers as this would have a better chance of success rather than adopting templates from the Global North which has historically failed in the Global South to achieve the purpose why they were implemented in the first place. Much of what has been referred to as the “New International Economic Order” has not addressed the peculiarities of the Global South (Deforge & Lemoine, 2021).

Furthermore, it is not enough to adopt legal frameworks from peers. Steps must be taken to ensure that unique Global South frameworks that have succeeded in places where they have been tried out do not fail when adopted by their peers. As a corollary, Lee notes that the “success of this legal adoption would depend on whether the cited laws could operate effectively under the different legal frameworks, institutions, and socioeconomic conditions in those countries” (Lee, 2017). Lee (2017) coins this as adaptability to socioeconomic conditions. It follows that developing economies must look at what their peers are doing to succeed. Also, it is critical that developing economies move beyond reforms to implementation. For example, the problem with SSA is not in law-making. SSA produces some of the most beautiful laws that can be found around the world (Natural Resource Governance Index, 2018). It is noted that the “benefits of a robust legal framework materialise only when institutions and practices to implement the rules are in place” (Natural Resource Governance Index, 2018). Countries must take positive steps to, among others, enforce existing laws that protect the rights of citizens and ensure that the rule of law is paramount as well as “improve the health of democratic institutions” (Transparency International, 2019). This postulation is easily referred to as “political will” (Lee, 2017).

## Conclusion and Recommendations

Extreme poverty should not only be seen as an economic problem or one that requires only economic solutions. The overarching discussion in this article is the role that law and the legal system can play in eliminating extreme poverty around the world and especially in the SSA region which has the highest concentration of people living in extreme poverty in the world. The article argues that the parameters used for assessing extreme poverty and extreme poverty based on economic patterns may not show the true picture of whether governments are doing enough or the right things to end extreme poverty. For example, basing the level of extreme poverty on a country’s GDP or using the poverty line to evaluate the performance of any given country may produce pyrrhic victories that do not reflect the true plight of vulnerable persons in society.

This article resonates with the view that extreme poverty is indeed a human rights violation. It supports the multidimensional approach for measuring and tackling the hydra-headed problem of extreme poverty around the globe. More importantly, the article evaluates three approaches to dealing with poverty. These are human rights approach that is centred on international human rights norms. Compliance with such international obligations can serve as a pedestal for eliminating extreme poverty. Secondly, the article evaluates an access-to-justice approach that is hinged on the rule of law concept. For example, a traditional “arbitration-styled” approach like what was obtainable in pre-colonial Africa is mooted in the article. A third approach evaluated in the article is the socio-economic conditions approach. This approach draws inspiration from Professor YS Lee’s adaptability to socioeconomic conditions theory. The theory supports the view that with the failure of Western neoliberal ideas in the Global South, countries of the Global South may do well to implement strategies that have been proven successful in countries with similar socioeconomic conditions. Notwithstanding, this writer argues that it is not enough to implement the socioeconomic conditions approach without the political will to ensure that any framework implemented is enforced.

The article recognises that the elimination of extreme poverty in the world is a task that must be done before the year 2030. It is therefore not a surprise that it is the first goal out of the 17 targets of the UN 2030 SDGs Agenda. Be that as it may, the article analyses data that shows that SSA accounts for the largest concentration of people living in extreme poverty compared to other regions of the world. As a corollary, much as it is a concern for the nations of the world to deal with extreme poverty, SSA has a critical role to play, if not a more important role.

This article recommends a critical action plan for all countries with a high concentration of people living in poverty. In this last decade of the UN’s 2030 SDGs Agenda, the governments in SSA especially may need to enforce policies to deal with the problem of extreme poverty. For countries going into elections in the short term, enforcing SDG Goal 1 via the suggested template described in this article, must be a feature of manifestos. The electorates must demand accountability from political officeholders by voting out parties without a clear plan to achieve this goal and by voting in leaders with pragmatic plans. Countries in the Global South should make constitutional provisions to require political actors to be accountable for the reduction of poverty. Where a president or governor fails to take steps to ensure that extreme poverty is eliminated, it should be considered an impeachable offence.

## Endnotes

^1^‘Poverty and the International Covenant on Economic, Social and Cultural Rights’ Statement adopted on 4 May 2001 by the Committee on Economic, Social and Cultural Rights (E/C 12/2001/10) para 3.

^2^Article 7 ICESCR.

^3^1979.

^4^1965.

^5^1966.

^6^1989.

^7^1990.

^8^2006.

^9^Resolution adopted by the General Assembly on 17 December 2018.
